# Haplotype-resolved genome assembly of poplar line NL895 provides a valuable tree genomic resource

**DOI:** 10.48130/forres-0024-0013

**Published:** 2024-04-23

**Authors:** Jie Luo, Yan Wang, Zihui Li, Ziwei Wang, Xu Cao, Nian Wang

**Affiliations:** 1 College of Horticulture and Forestry Sciences, Huazhong Agricultural University, Wuhan 430070, China; 2 Jiangsu Key Laboratory of Sericultural Biology and Biotechnology, College of Biotechnology, Jiangsu University of Science and Technology, Zhenjiang 212013, Jiangsu, China; 3 Key Laboratory of Silkworm and Mulberry Genetic Improvement, Ministry of Agriculture and Rural Affairs, Chinese Academy of Agricultural Sciences, Sericultural Research Institute, Zhenjiang 212013, Jiangsu, China

**Keywords:** Poplar, NL895, Genome assembly, Haplotype-resolved genome, Heterozygosity, Allele specific expression (ASE)

## Abstract

Poplar line NL895 can potentially become a model plant for poplar study as it is a widely cultivated elite line. However, the lack of genome resources hindered the use of NL895 as the major plant material in poplar. In this study, we provided a high-quality genome assembly for poplar line NL895 with PacBio single molecule real-time (SMRT) sequencing and High-throughput chromosome conformation capture (Hi-C) technology. The raw assembly of NL895 for the diploid genome included 606 contigs with a total size of ~815 Mb, and the monoploid genome included 246 contigs with a total size of ~412 Mb. The haplotype-resolved chromosomes in the diploid genomes were also generated. All the monoploid, diploid, and haplotype-resolved genomes showed more than 97% completeness and they can largely improve the mapping efficiency in RNA-Seq analysis. By comprehensively comparing the two haplotype genomes we found the heterozygosity of NL895 is much higher than other poplar lines. We also found that NL895 harbors more genomic variants and more gene diversity. The haplotype-specific genes showed higher variable gene expression patterns. These characters would be attributed to the high heterosis of poplar line NL895. The allele-specific expression (ASE) was also investigated and lots of alleles showed biased expressions in different tissues or environmental conditions. Taken together, the genome sequence for NL895 is a valuable tree genomic resource and it would greatly facilitate studies in poplar.

## Introduction

Poplar (*Populus*) is a fast-growing tree species that consists of ca. 30 species, and is widely distributed in the Northern Hemisphere. The relatively small genome size, ease of genetic transformation by *Agrobacterium,* and vegetative propagation make *Populus* a model for tree biology studies^[1]^. The first poplar genome *Populus trichocarpa* was released in 2006, to date, ca. 20 poplar genome resources have been released with the rapid updated sequencing and bioinformation methods, including *P. trichocarpa*^[2,3]^, *P. tremula*^[4,5]^, *P. tremuloides* Michx.^[5]^, *P. adenopoda*^[6]^, *P. lasiocarpa*^[7]^, *P. euphratica*^[8,9]^, *P. wilsonii*^[10]^, *P. alba* × *P. tremula* var. *glandulosa* (clone 84K)^[11,12]^, *P. deltoides* cultivar I-69^[13]^, *P. qiongdaoensis*^[14]^, *P. euphratica*^[15]^, *P. tomentosa* Carr.^[16]^, *Populus alba* '*Berolinensis'*^[17]^, *P. alba* var. *pyramidalis*^[18]^, *P. alba*^[19]^, *P. davidiana* Dode^[20]^, *P. pruinosa*^[21]^, *P. ilicifolia*^[22]^, *P. tremula* × *P. alba* (INRA 717-1B4)^[23]^, *P. simonii*^[24]^ and *P. koreana*^[25]^. The released poplar genomes uncovered the molecular mechanisms of extreme environmental adaptation, sex determination, biosynthesis of secondary metabolites, tissue development, and allopolyploidization effects of heterosis in poplar^[8−10,14,15,17,22,25]^.

Plants with high genomic heterozygosity usually potentially display heterosis, which endows plants with high productivity as well as strong abiotic stress resistance. Recently, several haplotype-resolved genome assemblies have been available for hybrid poplars^[11,12,16,18,23]^. For instance, the sequence differences of key genes involved in stress response between sub-genomes of *P. alba* var. *pyramidalis* contribute to the stress resistance^[18]^. The haplotype-resolved assemblies of *P. tremula* × *P. alba* uncovered the aspen-specific megabase satellite DNA PtaM147 repeats^[23]^. Furthermore, the phased assembly discovered that the transcriptional bias occurred between the two subgenomes of 84K (*P. tremula* × *P. alba*), the genes from subgenome *P. tremula* were dominantly expressed^[11]^. The low recombination ratio caused by the low sexual fertility has been found among the subgenomes of *P. tomentosa* may contribute to its high productivity and strong adaptation as 'fixed heterosis'^[16]^. However, the information about heterozygosity on genomic features in poplar need to be further explored.

Poplar NL895 (*P. deltoids* × *P. euramericana*) is a fast-growing hybrid poplar species that plays an important role in timber production and carbon sequestration in south China^[26]^. Due to the economic importance, lots of studies have been conducted with poplar NL895 to uncover the molecular mechanisms of adventitious root formation, nutrient uptake, growth, wood formation, callus formation as well as disease resistance^[27−33]^, which makes NL895 a potential model for genetic research on trees. Thus, a chromosome-scale, haplotype-resolved assembly of NL895 is urgently needed to facilitate the molecular studies in polar line NL895 and help understand the molecular basis for tree growth and development. Furthermore, the high heterozygosity of NL985 between the different haplotypes will shed light on the understanding of heterosis in woody plants. The raw assembly of NL895 for the diploid genome included 606 contigs with a total size of ~815 Mb and the monoploid genome included 246 contigs with a total size of ~412 Mb. The two haplotype genomes were also generated and both showed high quality. By using these genomic data, the heterozygosity of NL895 was comprehensively investigated. We found NL895 harbors more genomic variants, more gene diversity, and more variable gene expression patterns. These three characters would be attributed to the high heterosis of poplar line NL895. Taken together, the genome sequence for NL895 provided in this work is a valuable tree genomic resource and it would greatly facilitate studies in poplar.

## Materials and methods

### Plant materials

The poplar line NL895 was used as plant material in this study. NL895 is a cultivar generated through the cross between *P. deltoides* Bartr. cv. I-69 and *P.* × *euramericana* cv. I-45. Fresh leaves of tissue culture plants of NL895 were collected for DNA isolation and high-throughput chromosome conformation capture (Hi-C) library construction. For RNA preparations, different tissues including leaves, petioles, stems, shoots, and roots were also collected from tissue culture plants of NL895. DNA and RNA were isolated according to procedures reported in our previous studies^[34,35]^.

### Genome sequences generation

The pair-end (PE) reads were sequenced by using BGI-seq500 platform. The raw reads were filtered by using the software of Trimmomatic (version 0.39)^[36]^ with default parameters. Continuous Long Reads (CLR) were generated by using PacBio single-molecule real-time (SMRT) sequencing technology for NL895. Isoform sequencing (Iso-Seq) of mRNA for NL895 was also conducted by using the Single-molecule real-time (SMRT) PacBio platform. The long reads were called and filtered by using SMRT tools (version 9.0.0.92188) with default parameters. For comparison of genomic variants between NL895 and other poplar lines, public online data (Project ID: PRJNA687326) was retrieved from the SRA database. The PRJNA687326 project collected 103 poplar accessions and did whole-genome re-sequenced to uncover the phylogenetic relationships and biogeography history of *Populus*^[37]^.

### Kmer estimation, genome assembly and genome quality assessment

Kmer estimation was calculated with 'kmerfreq' implemented in GCE software^[38]^ with parameter setting as '-k 17'. Genome assembly of NL895 was performed by using several different software, including canu (Version: v1.9)^[39]^, hifiasm (Version: 0.19.5-r590)^[40]^, mecat2 (Update 20190304)^[41]^ and wtdbg (Version: 1.1.006)^[42]^ according the manual books of these pipelines. The best genome assembly was selected to polish using pilon^[43]^ (version 1.23) with default parameters. The polished genome assembly was then purged to generate the monoploid assembly by using the purge_dups pipeline (Version: 1.2.5). The un-purged genome assembly was regarded as diploid genome assembly. Pseudo-chromosomes were constructed for both diploid and monoploid by using Hi-C data and 3d-DNA (Version: 180922)^[44]^ and Juicebox (Version: 1.11.08)^[45]^ pipelines. Chromosome numbers were assigned according to sequence similarity with the *P. trichocarpa* genome^[2,3]^. Genome assemblies were assessed using Benchmarking Universal Single-Copy Orthologs (BUSCO)^[46]^ analysis (The lineage dataset: embryophyta_odb10, Creation date: 2020-09-10, number of species: 50, number of BUSCOs: 1614).

### Repeat sequence identification, gene prediction, and functional annotation

Repeat sequenced of the two sets of genomes were annotated with a combination of homology-based and *de novo* approaches. The software programs RepeatModeler 2.0, LTR Finder v. 1.0.6, and RepeatMasker v.4.0.5 were used to perform this analysis^[47−49]^. All parameters and pipelines were set according to our previous genome analyses^[47−49]^. The repeat-masked monoploid and diploid genomes of NL895 were used for gene prediction. Gene predictions were performed with three different strategies, including ab initio, homology-based, and transcriptome-based predictions. Briefly, AUGUSTUS (Version: 3.4.0)^[50]^ was used to perform the ab initio prediction. The Exonerate pipelines^[51]^ (Version: exonerate-2.4.0) were used to perform homology-based prediction. Proteins of five plants, including *A. thaliana*, *V. vinifera*, *O. sativa*, *P. trichocarpa* and *J. regia*, were used as the homology database for this analysis. RNA-Seq and Iso-Seq of NL895 were used to perform the transcriptome-based predictions with the Program to Assemble Spliced Alignments^[52]^ (Version: PASApipeline.v2.4.1). Finally, all three evidence of gene structure evidence was integrated with EVidenceModeler^[51]^ pipeline (Version: EVM1.1.1). Default parameters were set for all three analyses and these pipelines were identical to our previous genome analyses^[47−49]^.

### Gene expression analysis

A total of 60 RNA-Seq data generated from different tissues/treatments of NL895 in our previous studies^[31, 34]^ were collected for gene expression analysis. For mapping rate comparisons of RNA-Seq data among genomes of *P. trichocarpa*^[2,3]^, the monoploid and diploid NL895. Clean RNA-Seq was mapped onto these three genomes using Hisat2 with default parameters^[53]^. For allele-specific expression (ASE) analysis, clean RNA-Seq data was mapped onto the diploid genome of NL895 by using Hisat2^[53]^ pipeline with setting mismatch as zero. Differential gene expression and reads per million mapped reads (RPKM) analyses were performed using edgeR (Version: 4.3)^[54]^ software with default parameters.

### Genomic variants identification

For SNP/Indels identification among accessions, a standard GATK (Version: 4.2.2.0)^[55]^ pipeline was employed for SNP/Indel calling for different poplar lines. Briefly, PE reads from PRJNA687326 of 103 poplar accessions was mapped onto the 'A' chromosomes of the diploid genome of NL895. PE reads of NL895 were also employed in this analysis. PCR duplications of PE reads in each sample were then marked and removed. Subsequently, SNPs for each sample were identified. Finally, SNPs/Indels were filtered with the parameters 'QUAL <30.0 || QD < 2.0 || FS > 60.0 || SOR > 4.0'.

For identification of SNP/Indels between the two haplotypes of the NL895 diploid genome, an alignment between each chromosome pair was first performed and variants including SNPs and InDels were called in the collinearity blocks. Briefly, the two haplotype genomes of NL895 were first aligned with mummer (Version: 4.0.0)^[56]^ pipelines with parameters of '-l 15000 -i 80 -o 80'. Then, the best alignment for the two haplotypes were identified. Finally, the program 'show-snps' was implemented in mummer (Version: 4.0.0) and used to generate SNP/Indels between the two haplotypes.

### Gene pairs identification and structure variations (SVs) analysis

For gene pair analysis, the two haplotype genomes of NL895 were first aligned with mummer (Version: 4.0.0)^[56]^ pipelines with parameters of '-l 15000 -i 80 -o 80'. A reciprocal blast was also performed for proteins of the two haplotype genomes. A pair of genes were defined as alleles according to their locations and similarity in the 19 chromosome pairs if two genes showed the top similarity to each other and they were also located in a collinearity block. Structure variations (SVs) between the 2 haplotypes of the NL895 genome were identified by using MUMandCo (Version: 3.8)^[57]^ pipelines with default parameters.

## Results

### High-quality of genome assembly for poplar hybrid NL895

To predict the heterozygosity rate and genomic size of poplar hybrid NL895, a total of 51.22 Gb genomic clean pair end (PE) reads were generated (Supplemental Table S1). The distribution of 17-bp Kmer clearly showed there were at least two peaks, at 55- and 113-folds coverage (Supplemental Fig. S1). The second peak was much lower than the first one indicated a high heterozygosity rate of the genome. According to the algorithm reported in the software of GCE (Genome Characteristics Estimation)^[38]^, the heterozygosity rate of NL895 was calculated as 2.47%. The monoploid genomic size of NL895 was also predicted and it was 441 Mb. This predicted genomic size is similar to that of *P. trichocarpa* (446 Mb)^[2]^.

**Figure 1:**
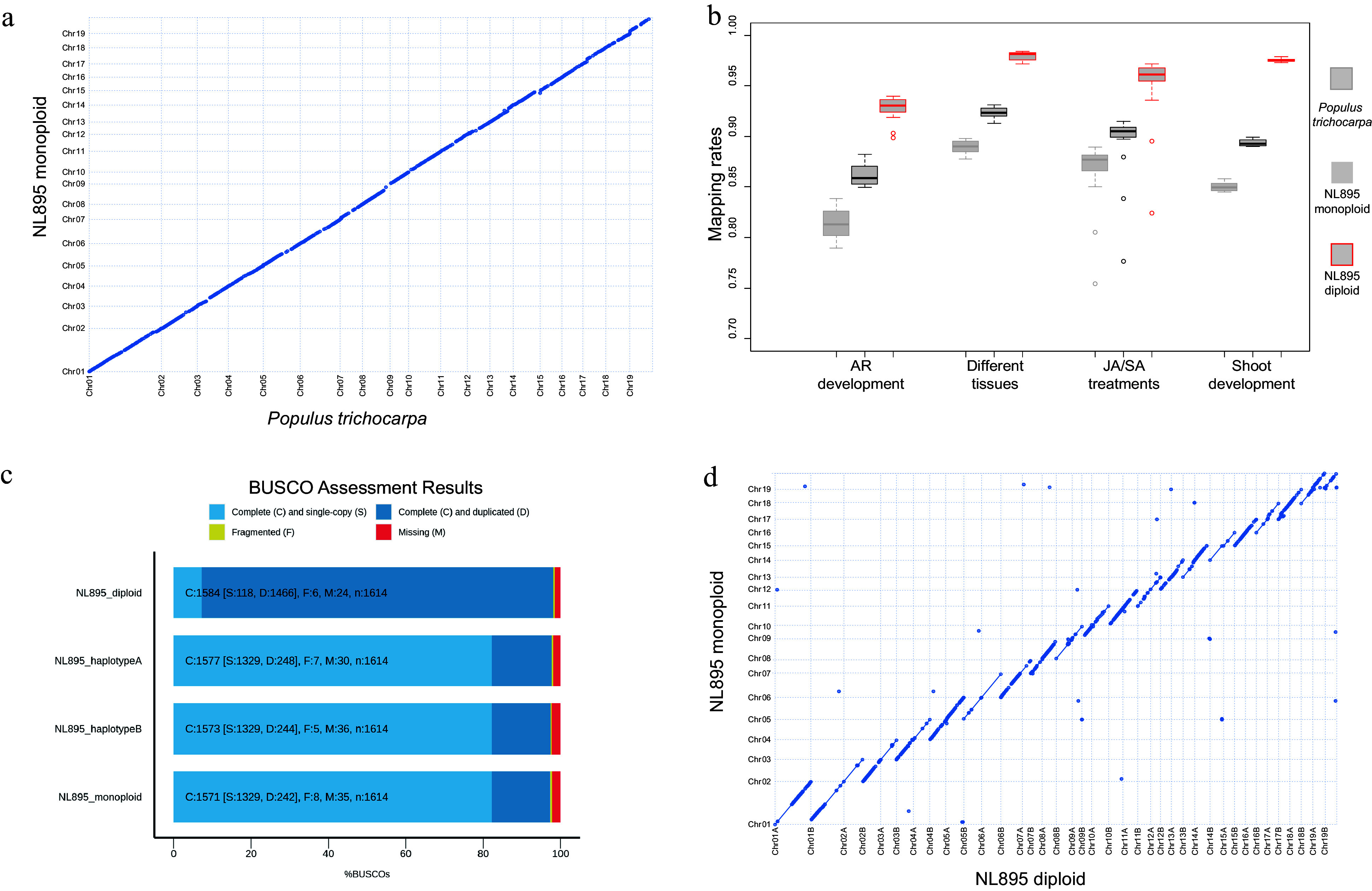
Assessment of genome assembly of poplar line NL895. (a) Collinearity between the genome of monoploid NL895 and *Populus trichocarpa.* (b) Mapping rate of RNA-Seq when the three genomes were used as reference. Four experiments including 60 RNA-Seq samples were used in this analysis. (c) Busco assessment for monoploid, diploid, haplotype A and B of the NL895 genome. (d) Collinearity between the genomes of NL895 monoploid and diploid.

To produce a high-quality genome assembly of NL895, a total of 159.14 Gb long reads were produced by PacBio single-molecule real-time (SMRT) sequencing technology. These data equaled to 360-fold coverage of NL895 genome size. These data included 9,459,789 reads and the N50 and average size of the clean reads were 26,047 and 16,823 bp (Supplemental Table S1), respectively. These long reads were assembled by widely-used software including canu^[39]^, hifiasm^[40]^, mecat2^[41]^ and wtdbg^[42]^ and the canu package was selected finally because it could produce the longest N50 and less contigs (Table 1 & Supplemental Table S2). The raw assembly of NL895 genome included 606 contigs with a total size of ~815 Mb (Table 1). This genome size was nearly double the predicted 441 Mb. The expanded size of the NL895 genome in the raw assembly could be attributed to its high heterozygosity. Then the genome assembly was polished and corrected by the 51.22 Gb genomic PE reads. Thereafter, two strategies were employed to generate monoploid and diploid genome sequences of NL895. First, the corrected genome assembly was purged and it produced the monoploid genome that included 246 contigs with a total size of ~412 Mb (Table 1). The sizes of N50 contig and the longest reached ~14.8 Mb and ~26.0 Mb, respectively. To construct chromosomes for NL895 genome assembly, Hi-C technology was employed to provide the orders and orientations for contigs in the 412 Mb genome assembly. In total, ~61.00 Gb clean PE reads were generated by BGI-Seq 500 sequencing platform for one Hi-C library that was constructed for NL895 DNA (Supplemental Table S1). A total of ~393.0 Mb sequences were grouped into the 19 chromosomes with an anchoring rate of 95.4% and the left sequences were merged into 117 scaffolds. The chromosome numbers were assigned according to sequence homology to *P. trichocarpa* genome. The largest chromosome was chromosome 1 with 48.0 Mb and the shortest chromosome was chromosome 12 with 13.7 Mb. To assess the genome assembly of NL895, the 19 chromosomes were aligned to the genome of *P. trichocarpa*. In a general view of this alignment, the two genomes showed very high collinearity (Fig. 1a). In total, there were 1,015 collinearity blocks with genomic size above 15 kb in the whole NL895 genome. The average sizes for these >15 kb collinearity blocks were 44.7 kb. The 19 chromosomes were aligned to the genome of *P. deltoides* I-69^[13]^, which is one parent of NL895. There were 3,293 collinearity blocks with genomic size above 15 kb in the whole NL895 genome. The average sizes for these > 15 kb collinearity blocks were 59.8 kb making a total size of 197 Mb. Unfortunately, there was no genome resource available for the other parent *P.* × *euramericana* cv. I-45. However, the result still suggested that the genome of NL895 showed higher similarity with its female parent than the genome of *P. trichocarpa*, however, there are still many variations between the genomes of NL895 and I-69.

**Table 1:** Summary of poplar NL895 genome assembly and annotation.

Features	Monoploid	Diploid	Haplotype A	Haplotype B
Assembly length (bp)	412,628,918	815,138,040	N/A	N/A
Contig N50 Length (bp)	14,829,479	13,599,823	N/A	N/A
Shortest sequence length (bp)	2,548	2,548	N/A	N/A
Longest sequence length (bp)	26,037,430	26,037,430	N/A	N/A
Total number of contigs	242	606	N/A	N/A
Final genome size (bp)	404,381,870	824,114,582*	377,691,676	378,545,799
GC content (%)	34.38	33.91	33.49	33.47
Protein-coding gene number	49,520	88,687	41,561	41,660
Transcript number	61,532	101,353	48,090	47,169
Average of gene length (bp)	3,218.7	3,150.6	3,231.7	3,188.5
Average of mRNA length (bp)	1,860.1	1,728.8	1,771.5	1,16.2
Average of CDS length (bp)	1,329.3	1,349.2	1,359.6	1,359.4
Exon number	321,605	519,987	252,863	244,402
Intron number	260,073	418,634	204,773	197,233
Average of exon length (bp)	355.9	337	336.9	331.2
Average of intron length (bp)	415.7	406.8	408	408.2
* This size includes ca. 67 Mb bp scaffolds that could not be assigned onto any haplotypes, below parameters were concluded according to 791,466,253 bp.				

A total of 60 RNA-Seq samples reported in our previous studies were also used to map onto the genome of the NL895 monoploid and *P. trichocarpa* with the same parameters (Supplemental Table S3). The mapping rate for NL895 monoploid ranged from 77.68% to 91.30%, while the mapping rate for *P. trichocarpa* ranged from 75.41% to 89.82% (Fig. 1b). The average mapping rate for NL895 monoploid was 89.33% and it was significantly higher than 85.51% for *P. trichocarpa* (*p *< 0.05, student's t-test) (Supplemental Table S4). The 412 Mb NL895 monoploid genome was also assessed by Benchmarking Universal Single-Copy Ortholog (BUSCO) pipeline (BUSCO version 4.1.4). There were 1,571 complete BUSCOs among the 1,614 searched BUSCO groups, including 1,329 and 242 single-copy and duplicated BUSCOs, respectively. There were also 8 and 35 fragmented and missing BUSCOs. The 1,571 complete BUSCOs indicate the completeness of the NL895 genome equaled 97.3% (Fig. 1c).

Secondly, the 815 Mb raw assembly of NL895 was also anchored by Hi-C data. The chromosome-scale diploid genome could be easily generated through Hi-C technology due to the high heterozygosity of the NL895 genome. A total of ~756 Mb sequences were anchored to 38 chromosomes. The chromosome numbers were assigned according to sequence homology to the monoploid genome and a suffix of 'A' or 'B' was followed with the chromosome number to differentiate the two chromosomes in one pair (Note: A or B were assigned randomly). Although there were ca. 67 Mb sequences that could not be assigned to the 38 chromosomes, the Hi-C interact heatmap could still show a high quality of chromosome clustering. Each of chromosome pairs also showed weaker interaction (Supplemental Fig. S2). Including the 38 chromosomes, there were a total of 697 scaffolds within the final NL895 diploid genome. These 38 chromosomes were aligned with the 19 chromosomes of the NL895 monoploid genome. The high collinearity could be observed between the chromosome pair and their corresponding chromosome in the monoploid genome (Fig. 1d). Moreover, collinearity blocks with 100% identity to the corresponding chromosome in the monoploid genome were also observed (indicated with lines but not dots). The total size of these blocks in each chromosome pair could almost cover the whole size of their corresponding chromosome and it suggested that the purged steps during the generation of monoploid genome usually retained one allele in each locus. This also indicated the monoploid genome information would lose some genome information and it is necessary to obtain a haplotype-resolved for plants with high heterozygosity. The 60 RNA-seq samples were also mapped onto the diploid genome and the mapping rate ranged from 82.39% to 98.42% (Supplemental Table S4). The overall average mapping rate was 95.91% and it was much higher than the monoploid and *P. trichocarpa* genome (Fig. 1b). BUSCOs search indicated that the diploid genome had 98.1% completeness with 7.3% (118 terms) single and 90.8% (1,466 single terms) duplicated BUSCOs (Fig. 1b), respectively. BUSCOs search of all 'A' suffix chromosomes showed 97.7% completeness with 82.3% (1,329 terms) single and 15.4% (248 terms) duplicated BUSCOs, respectively (Fig. 1c). The 'B' suffix chromosomes showed 97.4% completeness with 82.3% (1,329 terms) single and 15.1% (244 terms) duplicated BUSCOs, respectively (Fig. 1c). The high BUSCOs completeness for all three datasets suggested the diploid genome covered the most genomic information of hybrid NL895.

### Genome annotation

**Figure 2:**
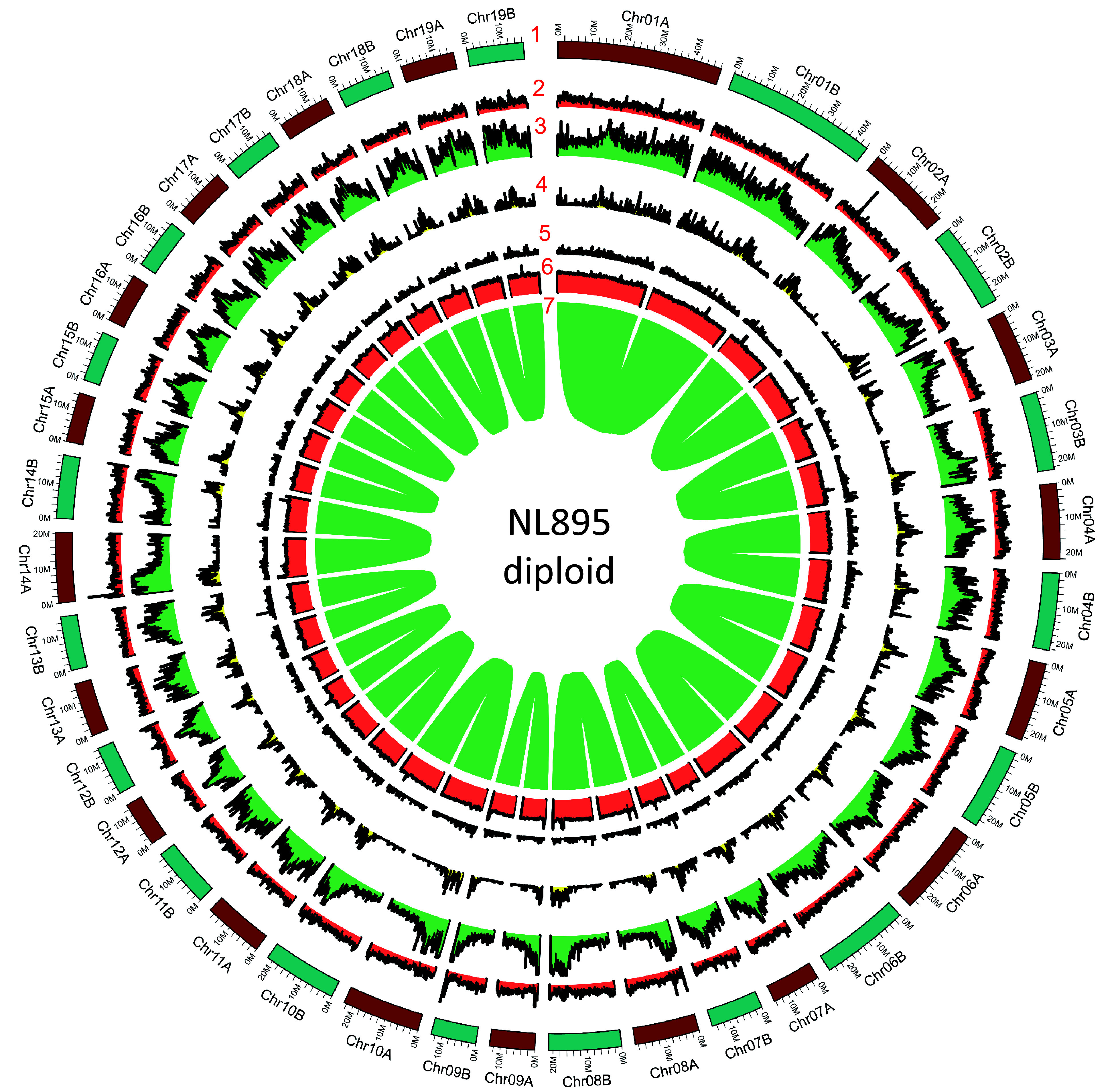
Genome annotation for the NL895 diploid. Numbers 1 to 7 represent chromosomes, gene density, repeat content, Gypsy transposon content, Copia transposon content, GC content and gene pairs, respectively.

Homology-based annotation and a *de novo* approach were applied to identify repeat sequences in the monoploid and diploid NL895 genomes. There were 46.95 % (189.8 Mb) and 46.49% (382.9 Mb) sequences were masked as non-redundant repeat sequences for monoploid and diploid, respectively (Table 2). Of these 46.95% sequences in monoploid, the proportion of long interspersed nuclear elements (LINEs), long terminal repeat (LTR), DNA transposon, rolling-circles, unclassified repeats, small RNA and satellites were 1.04%, 14.12%, 3.45%, 0.21%, 26.94%, 0.25% and 1.14%, respectively (Table 2). Similarly, the proportion of LINEs, LTR, DNA transposon, rolling-circles, unclassified repeats, small RNA and satellites were 0.96%, 13.69%, 3.35%, 0.20%, 26.82%, 0.25% and 1.18%, respectively (Table 2). All these numbers between the monoploid and diploid NL895 genomes are similar. To compare the above genomic characteristics between chromosome pairs in the diploid genome, their average parameters in each 1 Mb windows were calculated and illustrated in Fig. 2b. Insight of each parameter, the distribution showed similarities between chromosome pairs in each pair.

**Table 2:** Repeat sequences of the poplar NL895 genome.

Type	Monoploid				Diploid		
	Number	Length (bp)	Percent (%)		Number	Length (bp)	Percent (%)
LINEs	4,935	4,193,674	1.04		9,572	7,934,717	0.96
LTR elements	82,685	57,109,054	14.12		167,625	112,791,927	13.69
DNA transposons	18,295	13,945,655	3.45		37,091	27,637,781	3.35
Rolling-circles	1,504	869,410	0.21		2,934	1,669,040	0.2
Unclassified	314,000	108,124,463	26.74		647,665	221,061,595	26.82
Low complexity	20,817	992,939	0.25		43,019	2,076,941	0.25
Simple repeats	118,555	4,603,428	1.14		242,702	9,687,811	1.18
Total	560,791	189,838,623	46.95		1,112,622	382,859,812	46.46

To predict protein-coding genes for the NL895 genome, three strategies, including transcriptome-based, homology search, and ab initio prediction, were employed. A total of 49,520 genes nonredundant protein-coding genes with 61,532 transcripts were predicted for the monoploid genome (Table 1), while there were 88,687 and 101,353 genes and transcripts were predicted for the diploid genome (Table 1), respectively. The other gene characteristics, including gene length, exon length and intron length were similar between the diploid and monoploid genomes (Table 1). Functional annotation of all genes was performed by searching the public databases (InterPro, Nr, TrEMBL, and TAIR), and 47,195 (95.3%) and 84,251 (95.0%) genes showed at least one functional annotation in the monoploid and diploid, respectively. All these 49,520 genes in the monoploid genome were then assessed by the BUSCO pipeline and the result showed that the completeness of all NL895 predicted genes equaled to 94.9% with 10.5% duplicated BUSCOs. This number was just slightly lower than the completeness of the whole genome. Similarly, the 88,687 genes showed 98.1% completeness of BUSCOs with 7.3% and 90.8% single and duplicated terms, respectively. In summary, all the above data suggests that the gene prediction of the NL895 genome was acceptable and reliable. Additionally, the gene density for each chromosome pair was also calculated and illustrated in a 1 Mb window and the distribution patterns were very similar in most regions (Fig. 2).

### NL895 diploid genome harbors more genomic variants

**Figure 3:**
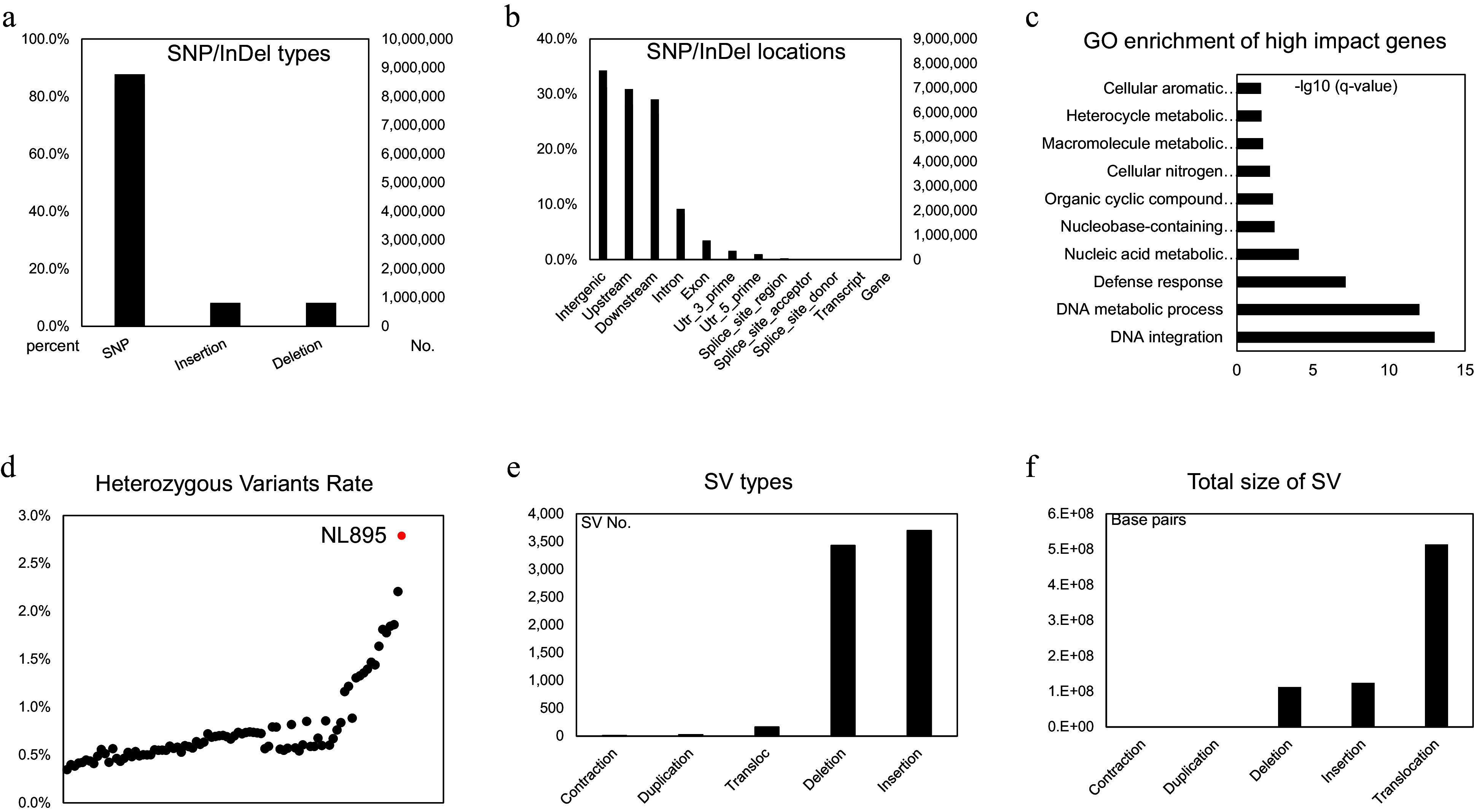
Genomic variants analysis in the diploid genome of NL895. (a) Numbers of SNP/Indels in the diploid genome of NL895. (b) Locations of SNP/Indels in the diploid genome of NL895. (c) GO enrichment of high impact genes by SNP/Indels. From the bottom to top, the full descriptions of GO terms are 'DNA integration DNA metabolic process', 'Defense response', 'Nucleic acid metabolic process', 'Nucleobase-containing compound metabolic process', 'Organic cyclic compound metabolic process', 'Cellular nitrogen compound metabolic process', 'Macromolecule metabolic process', 'Heterocycle metabolic process' and 'Cellular aromatic compound metabolic process'. (d) Heterozygous Variants Rates between poplar NL895 and other lines. The heterozygosity of NL895 is marked in red. (e) Numbers of structure variations (SVs) in the diploid genome of NL895. (e) Genomic sizes of structure variations (SVs) in the diploid genome of NL895.

NL895 is a highly heterozygous line with greater performance than other poplar lines and this phenomenon indicates this woody perennial plant harbors strong heterosis. The high heterozygosity of its genome would result in this strong heterosis. Therefore, the genomic variation between each chromosome pair was investigated by utilization of its high-quality haplotype-resolved genome sequence. To this end, an alignment between each chromosome pair was first performed and variants including SNPs and InDels were called in the collinearity blocks. In total, 10,406,261 variants were detected in the diploid genome and this took 2.76% proportion of the whole genome size (Supplemental Table S5). The percentage among chromosome pairs ranged from 2.49% to 3.04%. The average SNPs/Indels rate was 2.76% and it was slightly higher than the Kmer estimation (2.47%, Supplemental Fig. S1). Of the 10,406,261 variants, there were 8,771,356, 818,926 and 815,979 SNPs, insertions and deletions when the 'A' chromosomes were used as reference (Fig. 3a), respectively. Based on the gene locations, all these variants could be classified into 12 groups, including downstream, exon, gene, intergenic, intron, splice site acceptor, splice site donor, splice site region, transcript, upstream, UTR 3 prime and UTR 5 prime (Fig. 3b & Supplemental Table S6). Variants in intergenic, upstream, downstream regions of genes took the top three parts with 31.23%, 28.17% and 26.45% (Supplemental Table S6), respectively. When insight into the effect of these variants, a total of 28 types could be assigned by using the software of snpEff and their corresponding information was listed in Supplemental Table S7. The detailed information is listed in Supplemental Table S8. Some effects, such as frame shift, and gain or lost stop code, could largely affect the functions of genes. There were 11,944 genes that had at least one high-impact variant on their sequence and it took 28.74% part of genes in all 'A' chromosomes (Supplemental Table S8). A functional analysis of these 11,944 genes showed they tend to enrich in 'Cellular aromatic compound metabolic process', 'Heterocycle metabolic process', 'Macromolecule metabolic process', 'Cellular nitrogen compound metabolic process' and 'Organic cyclic compound metabolic process' (Fig. 3c). Most of the enriched biological processes related to energy metabolic pathways might suggest that the SNP/InDels variations in these genes would enable NL895 to possess the ability of high energy production.

To investigate the number of heterozygous SNPs/InDels between NL895 and other poplar lines, an investigation of genomic variants was conducted using public online data (Project ID: PRJNA687326). In that data, whole genome resequencing was performed for 88 poplar lines. To avoid variant calling affected by different methods, all 88 datasets and PE reads of NL895 were used to conduct a standard variant calling pipeline. In total, 58,434,834 SNP/InDels were found when the 'A' haplotype genome of NL895 was used as a reference for all 89 samples. The numbers of heterozygous variants in each line range from 1,315,099 to 10,535,490 and NL895 showed the largest number of heterozygous variants (Supplemental Table S9 & Fig. 3d). Interestingly, the largest number of these 88 lines was 8,338,955 and this number was still much less than NL895. The heterozygosity (heterozygous variants of NL895/genome size of haplotype A) of NL895 was 2.79% and this data was very consistent with the heterozygosity calculated by using the whole genome comparison or Kmer estimation. According to this analysis, it could be summarized that the NL895 genome harbored more genomic variants than other poplar lines.

SVs that showed genomic variation greater than 50 bp in the genome can usually generate large effect on gene features^[58,59]^. To identify SV between the two haplotypes of the NL895 diploid genome, MUMandCo pipelines were applied and a total of five types of SVs were detected, including contraction, duplication, translocation, deletion, and insertion. The numbers of these five types were 12, 26, 3,429, 3,697, and 163 (Fig. 3e), respectively. They covered 2,833, 56,258, 112,640,545, 123,948,305, and 513,703,830 bp genomic regions (Fig. 3f), respectively. Insight into these five types of SVs, contraction, and duplication took a very small part of genomic regions, while translocations might could be caused by wrong assembly of the genome (e.g., wrong placement of contigs during HiC scaffolding). Therefore, only deletion and insertion, also regarded as present and absent variation, were considered for further analysis. According to the location of predicted genes, a total of 50,636 genes harbored at least one deletion or insertion within its genomic sequence. Of these 50,636 genes, 2,168 and 26,225 genes only had deletion and insertion, respectively, while 22,243 genes had both types of SVs. This number took 60.85% parts for genes predicted on 38 chromosomes and it suggested that heterozygous SVs affected features of a large set of genes.

Taken together, the diploid genome of NL895 harbors lots of variants between the two haplotypes that are inherited from its parents, these variants include SNP/InDels, SV, and PAVs. There are many genes affected by these variants in their structures. Some of the affection in gene structures could largely change their functions and also create more gene diversity in the hybrid genome. The above data also revealed that the genome of NL895 harbors more variants than other poplar lines and this would cause strong heterosis for NL895.

### Haplotype-specific genes are shorter and expressed in lower levels

In the diploid genome, gene pairs could be located on homologous chromosomes with similar coordinates and the two genes were considered as alleles. To investigate how heterozygosity affects alleles in the diploid genome of NL895, alleles in the NL895 genome were first identified. A pair of genes were defined as alleles according to their locations and similarity in the 19 chromosome pairs. If two genes showed the top similarity to each other and they were also located in a collinearity block, these two genes are alleles. The collinearity block also should be the largest one where the two genes are located. According to these criteria, a total of 29,806 alleles were identified on the 19 chromosome pairs. There were ~67 Mb sequences that could not be assigned onto chromosomes in the diploid genome assembly, thus, some genes could have their alleles in the unanchored scaffolds. Therefore, a reciprocal blast for the left genes on 'A' and 'B' chromosomes or scaffolds to genes on scaffolds was performed and if two genes showed the top similarity to each other were also considered as alleles. This strategy enabled identification of 1,086 more alleles and the total number of alleles in the NL895 diploid genome was increased to 30,892 (Supplemental Table S10). There were 88,687 predicted genes in the diploid genome, thus, the 30892 alleles (61,784 genes) took 69.67% of all genes. The remaining 26,093 genes could be considered as haplotype-specific genes in NL895 diploid genome.

**Figure 4:**
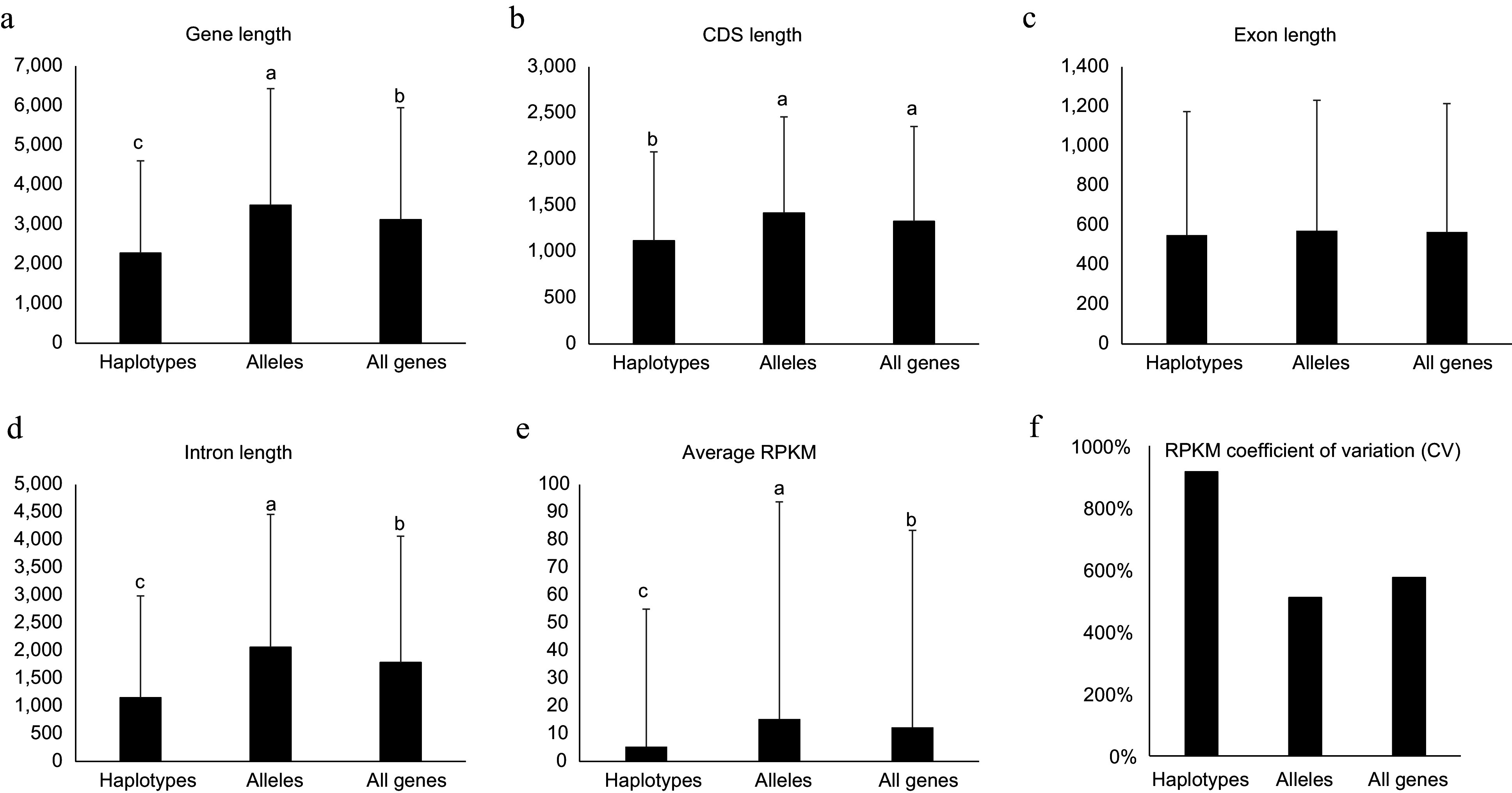
Genomic features for haplotype-specific, allelic and all genes in the diploid genome of NL895. Length of Genes, coding DNA sequence (CDS), exon and intron are shown in (a)−(d), respectively. The average of reads per million mapped reads (RPKM) and RPKM coefficient of variation (CV) are shown in (e) & (f), respectively. Multiple comparisons were performed for the average values among different groups with 'TukeyHSD' implemented in R in (a)−(e). Different lowercase letters indicate significant differences (*p* < 0.05). Error bars represent ± SD.

When comprehensively investigating gene features of the two types of genes, alleles and haplotype-specific, their gene length showed significant difference (*p* < 0.05, student's t-test). The average gene length for haplotype-specific, alleles and all genes were 2,273.2 ± 2,334.1, 3,484.6 ± 2,947.2, and 3,117.1 ± 2,830.9 bp (Fig. 4a), respectively. This data suggested that the length of haplotype-specific genes is much shorter than alleles. Then, the length of CDS, exon, and intron were also investigated. The average CDS for haplotype-specific genes was ca. 300 bp shorter than alleles and the intron for haplotype-specific genes was ca. 900 bp shorter than alleles (Fig. 4b & d). However, the average length of exon between haplotype-specific genes and alleles did not show significant difference (Fig. 4c). These data suggest that haplotype-specific genes had fewer exon numbers and shorter intron resulting in shorter gene length.

Additionally, the expression levels between haplotype-specific genes and alleles were also compared. A total of 60 RNA-Seq data including four experiments, adventitious rooting (AR) development, JA/SA treatment, shoot development, and different tissues, were employed for analysis (Supplemental Table S3). To avoid the confusion caused by homologous loci for the two alleles in one gene pair, each RNA-Seq read was mapped onto at most one locus. Interestingly, alleles (single genes in the gene pair) showed much higher RPKM values than haplotype-specific genes. The average RPKM for alleles was 15.4, while the average RPKM for haplotype-specific genes was 5.4 (Fig. 4e). To investigate the cause of critical different gene expression levels between the two types of genes, their 1.5 kb upstream promoter sequences were extracted and motif patterns were investigated. By searching the 469 motifs deposited in the new PLACE database (www.dna.affrc.go.jp/PLACE/?action=newplace), the two types of genes showed different motif patterns. Although the top five abundant motifs for both types of genes were identical, but the ranks after the top five were very different (Supplemental Table S11). When investigating of the number of promoters harboring at least one given motif, the ranks after the 38^th^ between the two types of genes also were very different (Supplemental Table S11). These data suggested that different motif patterns in the promoter regions could result in the critical different expression levels between haplotype-specific genes and alleles. Except for the gene expression level, the variations of gene expression among the 60 RNA-Seq samples were also calculated for haplotype-specific and allelic and all genes. The RPKM Coefficient of variation (CV) is much higher for haplotype-specific genes than alleles (Fig. 4f). RPKM CV for haplotype-specific and allelic and all genes are 916%, 510%, and 575%, respectively. This data suggested that the haplotype-specific genes showed much more varied expression patterns than alleles. Different tissues and samples treated with environmental conditions were collected for these 60 RNA-Seq samples. Thus, this data further suggested that the expression of haplotype-specific genes changed more in different conditions/tissues than in alleles.

### Structure variations (SVs) in promoter could not be the major cause for the allele-specific expression (ASE) patterns

**Figure 5:**
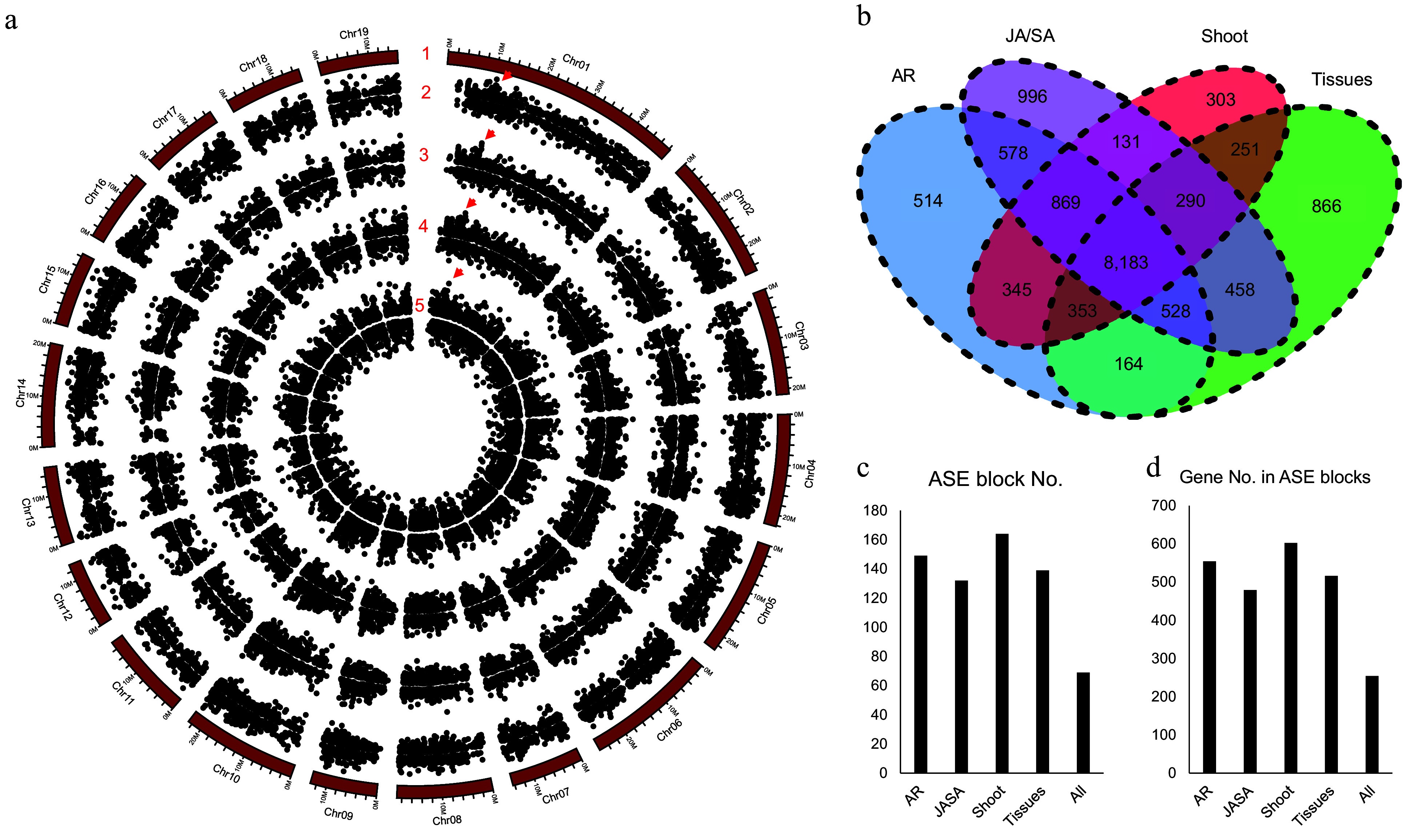
Allele specific expression (ASE) analysis for poplar NL895. (a) Number 1 represents chromosomes. Numbers 2−5 represent the locations of allele specific expression (ASE) in the four experimental data, different tissues (Tissues), JA_SA_Treatment (JA/SA), shoot development (Shoot), and adventitious development (AR), respectively. The largest ASE block was located on chromosome 1: 10,430,635−10,467,471 with ca. 36.8 kb in size and it is marked by red triangles. (b) Venn diagram of gene numbers of ASE in the four experiments. (c) ASE block numbers in the whole genome. (d) Gene numbers in the ASE blocks.

There are 30,892 allelic gene pairs in the diploid of NL895 genome and it takes 69.67% of all genes. Expression bias between the two genes of alleles is a common phenomenon. Thus, allele-specific expression (ASE) was analyzed by using the 60 RNA-Seq data. In total, there were 11,534, 11,093, 12,033, and 10,725 gene pairs showing ASE patterns under the criterion of log_2_FC < −1 or > 1 and false discovery rate (FDR) < 0.01. The ASE patterns on the chromosomes are summarized in Fig. 5a. With the removal of the redundancy of these genes, a total of 14,829 gene pairs showed ASE in at least one experiment. Among these 14,829 gene pairs, 8,183 pairs were showing ASE in all four experiments, and only 514, 996, 303, and 866 alleles showed ASE in one experiment (Fig. 5b). These data revealed that 55.18% (8,183/14,829) and 81.93% (12,150/14,29) alleles showed ASE in all four or at least two experiments (Fig. 5b). In other words, genes with ASE patterns are conserved in different experiments and these data might also suggest that the ASE patterns could be inheritable and it could be caused by some variations on genomic levels.

Since there were a lot of large heterozygous SVs existing in the NL895 diploid genome and large DNA variations usually could affect the expression of downstream genes, thus, the promoter sequence (2.0 kb upstream of start code) of all alleles was extracted and compared. There were 10,665 in all 30,892 gene pairs harboring SVs in at least one of the two alleles, while there were 5,052 in all 14,829 gene pairs that showed ASE patterns harboring SVs in at least one of the two alleles. The Fisher exact test indicated that SVs in the 2,000 bp promoter sequence could not be attributed to the ASE patterns for gene pairs (*p* = 0.50). Moreover, when insight into the 8,183 gene pairs that showed ASE patterns in all four experiments, a total of 2,447 gene pairs harbored SVs in their promoter regions. These data revealed that ca. 30% of gene pairs harbor SVs in their promoters, while also ca. 30% of gene pairs with ASE patterns harbor SVs in their promoters. These data also suggested that large fragments of insertions/deletions in the promoter regions in gene pairs could not be the major cause for the ASE patterns. In other words, all variants in the promoters of gene pairs including small insertions/deletions, SNPs, and SVs would be attributed to ASE in gene pairs.

Genes with ASE patterns were found located in linked genomic regions in some previous reports^[59]^. With insight into the locations of ASE on the 19 chromosomes, there were some ASE regions showing bias expression to one chromosome (Fig. 5a). A genomic region harboring more than three genes that were expressed higher or lower in one chromosome than the other was designated as an ASE block. In the four expression experiments, there were 149, 132, 164, and 139 ASE blocks with 554, 479, 602, and 516 genes for AR, JA/SA, shoot, and tissues (Fig. 5c & d), respectively. Moreover, with comparisons of all ASE blocks, a total of 69 ASE blocks were found to share in all the four expression experiments and these 69 ASE blocks harbored 254 genes. The largest ASE block was located on chromosome 1: 10,430,635−10,467,471 with ca. 36.8 kb in size and it harbored eight gene pairs (Fig. 5a). The eight genes on chromosome 1A showed higher expression than the expression of their alleles in chromosome 1B. According to the above analyses, it can be concluded that ASE is common in the diploid genome of NL895. The SVs could not be the major cause for the ASE and genes in some genomic regions showing expression bias in one chromosome.

## Discussion

The poplar line NL895 is an elite cultivar growing alongside the Yangtze River. Compared with other poplar lines, NL895 shows high heterosis. According to the genome analysis in this study, at least three evidence can explain the cause of heterosis. First, the genome of NL895 showed higher heterozygosity. Both Kmer and SNP analyses revealed that the heterozygosity of NL895 is ca. 2.75%. This number is much higher than an ordinary poplar line (Fig. 3d), as well as several other tree plants, such as 1.0% for walnut, 1.60% to1.62% for rubber tree and 0.79% for willow tree^[60−62]^. In our previous study, an elite cultivar of American grapes was also found to harbor ca. 2.70% heterozygosity^[63]^. Generally, individual plants with higher heterozygosity in a population would potentially show elite performance and higher heterosis^[64]^. However, high heterozygosity of polar genomes would bring deleterious mutations and genomic islands of divergence within a poplar species^[65,66]^. These phenomena would make NL895 not suitable to be used as parents for producing elite offspring. Therefore, as an elite cultivar, asexual propagation such as cottage would be the best strategy for generations of new plants with large numbers.

Second, higher gene diversity is in the NL895 genome. There are 88,687 genes in the diploid genome of NL895. Among these genes, 30,892 allelic pairs (69.67%) and 26,93 haplotype-specific genes were predicted. Usually, two genes in one allelic pair function identically or similarly. Thus, it indicated there were 56,985 (30,892 + 26,093) genes showing different functions. This number is much higher than 49,520 genes in the monoploid genome. More functional genes would bring heterosis to the poplar line NL895. Previously, phylogenomics of the genus *populus* reveals extensive interspecific gene flow and balancing selection^[67]^. Considering the genomes of parents of NL895 are also heterozygous and they would obtain functional genes from other populus species. This would further increase gene diversity in the genome of NL895.

Third, varied gene expression patterns of haplotype-specific genes allowed NL895 to adapt to different environments better. The higher ability of environmental adaptions usually could bring higher heterosis and this phenomenon has already been reported in some studies^[68,69]^. Varied gene expression could be the major driver for plant adaption^[70,71]^. In NL895, there are 26,093 haplotype-specific genes and their average RPKM is ca. 5.4 among the 60 RNA-seq samples. Although the relative expression levels are much lower than alleles, the CV of RPKM for these 26,093 haplotype-specific genes is much higher. This data suggested that these 26,093 haplotype-specific genes showed a much higher variation of gene expression than alleles. In other words, haplotype-specific genes showed very high expression in some environments, while they showed very low expression in some other environments. The large variations of gene expression would bring NL895 a higher ability for environmental adaptions. Taken together, the high heterosis of NL895 was uncovered according to a comprehensive analysis of its high-quality genome. Thus, the high-quality genome resource allows us insight into the heterosis in NL895.

The poplar line NL895 is mainly grown the South China. In this region, the spring and summer seasons are generally characterized by high temperatures or high humidity. Some other lines, such as lines 717 (*P. tremula* × *P. alba*), shanxin yang (*P. davidiana* × *P. bolleana*), *P. tomentosa,* and black cottonwood (*P. trichocarpa*), that widely used in poplar studies can not grow well in fields of South China. Poplar line NL895 shows elite performances in this region both in the field and laboratory. More importantly, this line can grow fast and be easily transformed. In the past decade, there were some studies conducted by using NL895 as the major plant material and this line can potentially become a model plant for poplar biology study^[27,28,30,34,35,72−78]^. However, the lack of genome resources hindered the use of this line. In this study, we provided the haplotype-resolved genome sequence for NL895 would greatly facilitate studies by using NL895 as the major plant material.

## Conclusions

In this study, we generated a high-quality of genome assembly for poplar hybrid NL895. Both monoploid and diploid genomes were released. By assessing these two-genome assemblies with different parameters, both showed high quality. By taking advantage of these genome resources, we found NL895 harbors more genomic variants, more gene diversity, and more variable gene expression patterns. These three characters would be attributed to the high heterosis of poplar line NL895. Taken together, the haplotype-resolved genome sequence for NL895 is a valuable tree genomic resource and it would greatly facilitate studies in poplar.

## SUPPLEMENTARY DATA

Supplementary data to this article can be found online.

## Data Availability

The whole genome sequence data, including PE short reads, CLR reads, Hi-C interaction reads, transcriptome data, and genome files, have been deposited in the NCBI under accession number PRJNA1061373. The genome information can also be retrieved from our tree genome database (http://tree-bio.hzau.edu.cn/download/NL895/V1).
