# Supplementary figures and images for: Haplotype-resolved genome assembly of poplar line NL895 provides a valuable tree genomic resource
Source: For Res (Fayettev). 2024 Apr 23;4:e015. doi: 10.48130/forres-0024-0013 (PMC11524272; doi:10.48130/forres-0024-0013)

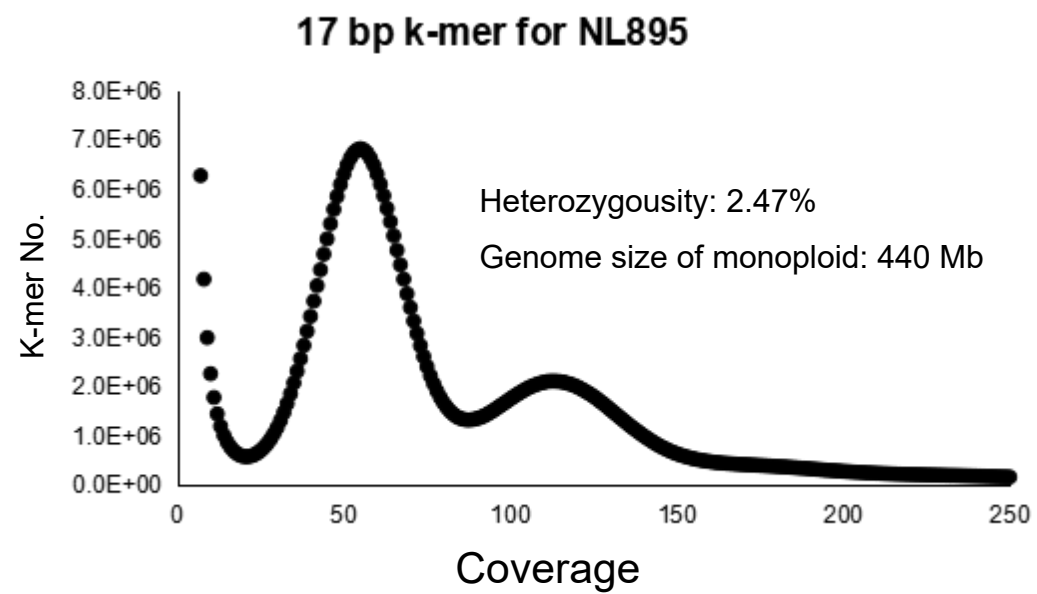

Fig.S1. Distribution of 17-bp Kmers

Supplement: Supplementary file 1 — Supplementary data to this article can be found online. [file forres-0024-0013-S1.zip › 10.48130_forres-0024-0013-Suppl-FigureS1.pdf]

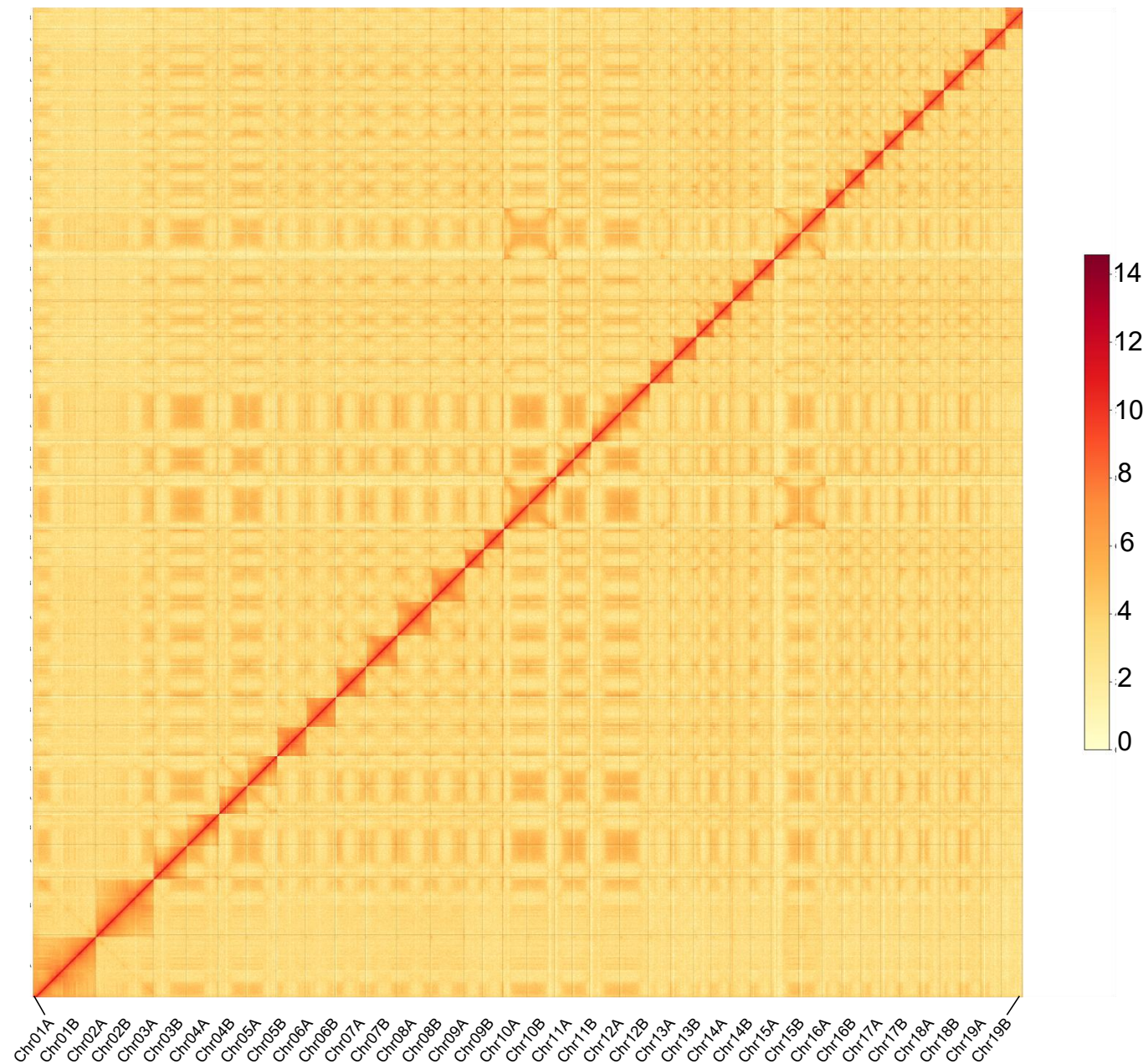

Fig. S2. Hi-C interaction matrix heatmap of NL895 diploid genome

Supplement: Supplementary file 1 — Supplementary data to this article can be found online. [file forres-0024-0013-S1.zip › 10.48130_forres-0024-0013-Suppl-FigureS2.pdf]
